# Comparative analysis of AI and expert evaluations in engineering design pedagogy

**DOI:** 10.1371/journal.pone.0332715

**Published:** 2025-09-22

**Authors:** Tuğra Karademir Coşkun, Esra Bozkurt Altan

**Affiliations:** 1 Computer Education, Faculty of Education, Sinop, Turkiye; 2 Science Education, Faculty of Education, Sinop, Turkiye; Massachusetts Institute of Technology School of Engineering, UNITED STATES OF AMERICA

## Abstract

**Background:**

Integrating engineering design processes into science education has become a significant priority in STEM instruction. However, many science teachers face difficulties incorporating these processes due to limited pedagogical expertise. Generative artificial intelligence (GAI) tools such as ChatGPT offer potential support mechanisms by evaluating lesson plans and providing formative feedback. This study investigates the reliability and validity of GAI evaluations compared to expert assessments.

**Methods:**

This mixed-methods study involved 43 science teachers who received professional development over four months to integrate engineering design into their lesson plans. A total of 52 lesson plans were evaluated using structured and unstructured prompts via ChatGPT 4.5, alongside evaluations by expert mentors. Quantitative data were analyzed using the Intraclass Correlation Coefficient (ICC) and Bland-Altman methods to assess inter-rater consistency. Qualitative data was analyzed through open and deductive coding to interpret differences in evaluation rationale.

**Results:**

Findings revealed high consistency between structured prompt AI evaluations and expert assessments (ICC = 0.708), while unstructured prompts showed low and non-significant agreement (ICC = 0.076). Qualitative analysis indicated that AI evaluations, particularly those using structured prompts, tend to be more positive and holistic, whereas experts offered more detailed and critical feedback. Differences were also observed in evaluating dcomponents like problem definition, testability, and interdisciplinary integration.

**Conclusion:**

Structured AI prompts offer reliable and valid evaluation results comparable to expert assessments and could serve as scalable tools in teacher support systems. However, unstructured prompts produce inconsistent outcomes and require refinement. The study highlights both the potential and limitations of using GAI tools for pedagogical evaluation in STEM education.

## Introduction

STEM education is widely recognized as one of the leading educational approaches of today, aiming to teach science, technology, engineering, and mathematics disciplines by integrating them with real-life problems [1]. The application of this approach in science education has become a significant area of interest for researchers in recent years [2,3]. In particular, the Next Generation Science Standards [4] and numerous other studies [5–8] emphasize the implementation of the STEM approach in science classrooms through the integration of engineering practices. Engineering is also regarded as an effective means to create a learning environment conducive to the STEM approach in science classrooms, as it often plays a unifying role with the other STEM disciplines [3,9]. This integration supports the development of 21st-century skills by enhancing students’ academic achievement in science courses, conceptual understanding [10,11], problem-solving [12,13], and creative thinking skills [14].

Interdisciplinary pedagogical approaches that aim to integrate engineering into science education for in-depth science learning are known by various terms such as design-based learning [14], design-based science [15,16], engineering epistemology-oriented learning [17] and science learning by design [18,19]. In this study, the term “design-based learning” (DBL) is employed. DBL, a prevalent approach in STEM education, incorporates the design processes utilized by engineers to address real-world problems into classroom practices [20]. To solve real-life problems, students must acquire scientific, mathematical, and technological skills. The DBL approach facilitates the application of students’ knowledge to complex problems, enhances their motivation, and improves their engineering acumen by immersing them in the design process [20–22].

While significant attention is devoted to the integration of engineering disciplines into science lessons in literature, numerous studies indicate that many science teachers lack sufficient experience in engineering practices or possess inadequate knowledge in this domain [23,24]. It’s important to stress that without enough support, teachers might avoid using engineering practices in their lessons, or they may not manage them well [25–27]. One of the primary obstacles in the implementation of the Next Generation Science Standards [28] in the United States is the inability of middle school teachers to address the engineering components of the standards [29–31]. To successfully integrate engineering disciplines into their classrooms, science teachers need to comprehend engineering pedagogy [32–34], examine classroom practices [35–38], and receive support for developing lesson plans or activities during professional development processes [39,40]. Consequently, the effective design and implementation of professional development programs are critical for teachers to successfully integrate engineering disciplines into their classrooms.

However, teacher support for integrating engineering disciplines into the classroom environment should not be limited to traditional professional development programs alone. In recent years, research on the potential of generative artificial intelligence (AI) tools to contribute to this process has been increasing. Kehoe (2023) examined how generative AI tools can facilitate lesson planning for pre-service teachers, noting that these tools contribute to teachers’ efficient use of time and provide structured content [42]. Yet, it was emphasized that teachers’ experience and expertise are needed for this content to form the basis for pedagogical decisions [41]. Moundridou et al. (2024) evaluated how generative AI tools can be classified in creating Inquiry-Based Learning lesson plans and assessed the potential contributions of these tools at each stage of teaching [42]. This is an example of systematically integrating AI into pedagogical processes [42]. Following these developments, a six-dimensional evaluation model (pedagogical, representational, communicative, scientific content, ethical, and transparency) has been proposed to more systematically assess the impact of generative AI in education. This model provides a comprehensive framework for how technology can be used more ethically, accurately, and effectively in education [43].

The use of generative artificial intelligence (GAI) tools in education can significantly impact teachers’ lesson planning processes by providing valuable support in various aspects such as curriculum design, assessment, and implementation. The benefits of GAI tools in lesson planning include increased efficiency in curriculum design, personalized learning experiences for students, and the ability to effectively address individual learning needs [43]. These tools can provide valuable insights to help teachers improve their instructional strategies. In addition to this, the capacity of generative artificial intelligence (GAI) tools to provide feedback on STEM lesson plans containing engineering components brings along some strengths as well as various limitations. For example, tools like ChatGPT can encourage critical thinking and increase student engagement by offering personalized feedback to students [44]. Teachers can more effectively observe and intervene in a timely manner on points where students struggle, thanks to multiple data streams generated by AI [45]. Moreover, these systems can make abstract engineering concepts more understandable through analogical thinking [40] and provide support to teachers in identifying professional competencies in STEM fields [46]. However, there are also potential disadvantages associated with GAI tools. It is observed that generative AI systems still have limitations in contextual understanding and providing social-emotional feedback [45]. Additionally, while providing excessive convenience to students may threaten academic integrity [44], bias and representation issues in educational data can affect the accuracy of content presented specifically for engineering [47]. Although generative artificial intelligence tools offer promising features, their applications for lesson plan evaluations, particularly in the context of STEM education, have not yet been sufficiently researched.

Recent studies have raised the question of whether generative AI tools can provide consistent and in-depth evaluations at the same level as human expert mentors. Some findings indicate high correlations between AI and human evaluators, especially in assessing structured written responses; yet, limitations have been reported in areas such as creative thinking and contextual interpretation [48]. In a study comparing artificial intelligence and teacher evaluations of university exams, moderate consistency was found in the evaluation of visual-based exams, low consistency in video exams, high consistency in test exams, and low consistency in traditional exams [49]. Therefore, it is stated that AI has not yet reached human mentor intuition and cannot achieve full pedagogical impact without comprehensive training and expert supervision. In this context, hybrid models combining AI and human expert evaluation are suggested as the most efficient approach in terms of both cost and consistency. In such a model, AI assumes the function of rapid and preliminary assessment, while experts review final decisions to ensure quality assurance [50]. While traditional mentoring requires significant human resources, generative AI tools can offer scalable and cost-effective alternatives; however, the pedagogical validity of these tools is not yet clear.

This research is structured according to three important gaps and needs in literature. Firstly, it is frequently emphasized that teachers lack sufficient knowledge and experience in integrating engineering disciplines into science courses; however, technology-based solutions to address this deficiency, especially generative artificial intelligence (GAI) supported interventions, have limited presence in literature. Secondly, there is a lack of systematic comparative studies examining the extent to which generative AI tools produce results that align with those of human experts in pedagogical evaluation. This gap is especially evident in the analysis and development of lesson plans. This situation reveals a serious research need in the context of GAI’s assessment validity, reliability, and depth. Thirdly, the pedagogical impact of prompt types used in interaction with GAI tools—structured and unstructured forms—on the generated outputs has not yet been sufficiently investigated. While the potential of structured prompts to increase content accuracy, consistency, and pedagogical quality is emphasized, empirical findings measuring this effect, especially in engineering design-based teaching processes, are quite limited.

### 1. Literature review

#### 1.1. Engineering pedagogy: design-based learning.

**Fig 1:**
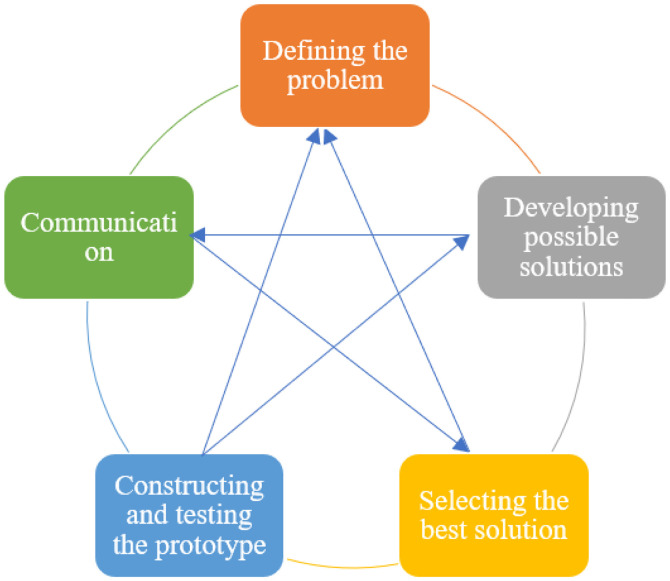
Engineering design process.

The engineering design process involves defining the problem, developing possible solutions, selecting the optimal solution, constructing and testing the prototype, and presenting the results (Fig 1).

These stages are cyclical and interactive [4,51–54]. During the problem identification stage, students are expected to define the problem and identify the criteria and constraints necessary for a viable solution [9]. This stage is crucial for developing alternative solutions and recognizing the requisite scientific knowledge. At the stage of presenting possible solutions, students propose various solutions by integrating the knowledge acquired through scientific research and inquiry into problem-solving [9,52]. At the stage of selecting the best solution, students engage in the optimization process typical in engineering by evaluating proposed solutions, designing, and testing prototypes [55]. Students then create the prototype for the best solution, refine it as necessary, and evaluate and present the entire process during the communication phase [9]. In these stages, scientific communication, research, and engineering design processes play a pivotal role [9,56]. Implementing and integrating all these stages into course outcomes demands significant expertise and dedication.

#### 1.2. Mentorship as a framework for teacher education in design-based learning.

Research into professional development programs designed to help teachers integrate engineering into their lessons typically examines the lesson plans they create and highlights the difficulties encountered along the way [35,57–59]. One significant challenge is effectively connecting engineering design tasks with science concepts. Studies show that teachers often prioritize teaching design skills and practices, leading to a reduced focus on engineering concepts for students [60]. Additionally, teachers frequently encounter difficulties in integrating problem definition, a critical initial stage of the engineering design process, into their lesson plans [61]. Furthermore, teachers may face barriers related to their design expertise and collaborative processes. The presence of teachers who lack the necessary knowledge and skills in collaborative design processes, such as working with experts and colleagues, can hinder the successful integration of engineering elements into curricula. Moreover, teachers may struggle to understand how to relate engineering design to other STEM subjects [62].

This underscores the necessity for comprehensive support systems to enhance teachers’ professional development for the effective implementation of engineering design-based education. High-quality professional development programs tailored for teachers across various grade levels can equip them with the requisite knowledge and skills to integrate engineering concepts into their lesson plans [63]. In this context, the mentoring model emerges as a valuable framework to support and guide teachers’ professional development. Mentoring is defined as the process in which an experienced expert aids the professional growth of a less experienced individual by sharing knowledge and experiences [64]. The literature includes numerous studies on mentoring programs aimed at providing professional development to teachers across various subjects [65,66]. Effective mentoring programs furnish teachers with essential guidance, support, and resources to navigate the complexities of the teaching profession [67]. Notably, long-term mentoring practices have proven effective in cultivating pedagogical content knowledge among science teachers [65]. Science mentor teachers can enrich the practice of novice teachers by offering valuable insights and professional development opportunities [68].

When it comes to integrating engineering design processes into lesson plans, having mentors in a 1:1 or 1:2 ratio creates an optimal environment for teacher learning and development [69]. However, the availability of mentoring support for teachers developing lesson plans often hinges on extensive research projects [59,70,71]. For instance, in their study, Zhu et al. (2018) were able to provide mentoring support for engineering practices to only 12 teachers nationwide [59]. In the current study, 43 science teachers are supported through the mentoring model to integrate engineering into science classrooms as part of a large research project. While the literature discusses the concept of peer mentoring among teachers, the sustainability of mentoring programs may be compromised if mentors take on these responsibilities without additional incentives or a reduced workload [72]. That simply appointing experienced teachers as mentors does not automatically meet the initial needs of novice teachers [71]. Expert mentors offer significant benefits, including enhancing professional skills, motivation, and overall performance [73]. Knowledgeable mentors can provide mentees with specialized insights and foster an active curiosity to improve their teaching practices [74]. However, challenges such as costs, sustainability, and the continuity of mentoring programs can impede the long-term success of these initiatives [75]. Moreover, ensuring continuity in the transfer of teaching practices from expert mentors to pre-service teachers in ratios such as 1:1 or 1:2 necessitates substantial expert resources. These challenges underscore the critical need for supporting teachers’ professional development for engineering integration, a necessity frequently highlighted in the literature [30,31,33].

At this point, it becomes inevitable to leverage the potential of technology to address the challenges in teachers’ professional development, facilitate one-on-one support, and provide a certain level of expertise. In particular, newly developed generative artificial intelligence (GAI) tools equipped with advanced language processing models can serve as new mentors for teachers. Indeed, recent research indicates that generative artificial intelligence (GAI)-supported teacher training significantly enhances teaching competence and higher-order thinking skills [76]. Furthermore, GAI tools demonstrate their capacity to offer substantial guidance in pedagogical content development, thereby enabling teachers to customize instructional materials with greater efficacy [77]. For teachers to utilize these tools efficiently, not only individual effort but also institutionally provided continuous professional development programs and infrastructural support are of critical importance [78].

#### 1.3. Prompt design and ıts ımpact on generative AI reliability.

The accuracy and reliability of the results produced by generative AI tools depend on the quality of the input data (NLP) and the robustness of the algorithms used. Structured prompts provide clear directions and hints, whereas unstructured prompts lack clear directions and follow a more open-ended approach to text generation [79]. The effectiveness of prompts in text production is critical, as the clarity of instructions can affect the quality and relevance of the produced text [80]. Structured prompts that specify performance criteria can lead to more accurate and consistent text production compared to unstructured prompts [80]. Furthermore, the design and objectives of the instruction interface can influence the creativity and coherence of the text produced by AI [81].

Recent research has further highlighted the pedagogical value of structured prompts in AI-assisted learning environments. For instance, the SMART prompt format—consisting of Seeker, Mission, AI Role, Register, and Targeted Question—has been shown to significantly enhance the accuracy, relevance, and source quality of AI-generated responses in medical education [82]. Similarly, engineering students who engaged with structured prompts demonstrated higher achievement in programming and data analysis tasks, suggesting these prompts facilitate greater cognitive focus [83]. Moreover, structured prompts have yielded more consistent outputs across sessions, as evidenced by higher Cohen’s κ scores compared to less detailed approaches [84]. By contrast, unstructured prompts have exhibited several limitations. These include lower consistency in AI responses [85], insufficient depth in fostering reflective thinking in educational settings [86], and increased risk of hallucinated or misleading answers due to lack of detail [84]. Empirical research comparing the effectiveness, reliability, and consistency of structured versus unstructured AI prompts in pedagogically supporting instructional activities within the context of engineering design processes remains limited.

### 2. Research aim and hypotheses

This study aims to examine whether generative artificial intelligence (GAI) based approaches produce valid, reliable, and consistent results in the evaluation of lesson plans that include engineering design processes, and to what extent these results overlap with expert mentor evaluations. Additionally, the study comparatively analyzes the effectiveness of structured and unstructured prompts in producing pedagogical evaluations. The sub-hypotheses of the related research are detailed below.

### H1: The consistency between structured prompt artificial intelligence evaluation scores and expert evaluation scores in the evaluation of teachers’ lesson plans enriched with engineering design process is significant

Hypothesis H1 aims to measure the degree to which the evaluation scores generated by structured prompts used by artificial intelligence (AI) tools in assessing teachers’ lesson plans—enriched with the engineering design process—align with the scores given by domain experts. In this context, structured prompts provide specific instructions that guide AI systems to evaluate teachers’ lesson plans according to predefined criteria. These criteria include the integration of engineering design processes, student engagement, achievement of learning objectives, and the effectiveness of pedagogical approaches. The AI system analyzes this data and assigns a score based on these criteria. Conversely, domain experts evaluate the same lesson plans based on similar criteria but incorporate human observation and evaluation. Expert evaluations may include subjective interpretations and deep expertise, potentially resulting in different outcomes compared to AI evaluations. Confirming Hypothesis H1 would support the efficacy of AI tools in aiding teachers to integrate engineering design processes into lesson plans and affirm the objectivity and reliability of these assessments. A high level of consistency between AI and expert evaluations would indicate that AI evaluation systems can serve as reliable tools in educational assessments. Conversely, a low level of consistency would suggest the need for improvements in AI evaluation processes.

### H2: The consistency between teachers’ unstructured prompt AI systems evaluation scores and expert evaluation scores in the evaluation of lesson plans enriched with the engineering design process is significant

This hypothesis aims to demonstrate a significant alignment between the evaluation scores produced by AI systems using unstructured prompts and the scores given by experts. Unstructured prompts provide AI systems with a free format, allowing for a broader scope of creative interpretation of the given lesson plan content. Such prompts enable the system to evaluate lesson plans without strict, predetermined criteria. Expert evaluations, typically conducted by experienced educators, assess how well teachers’ lesson plans adhere to educational standards and incorporate engineering design principles. These evaluations also consider pedagogical aspects, such as teaching methodology and student engagement. If confirmed, the proposed consistency in this hypothesis would suggest that AI systems can effectively evaluate lesson plans enriched with the engineering design process, potentially providing an affordable and accessible alternative for lesson plan evaluation, particularly in resource-limited educational settings.

### H3: There is a significant consistency between the scores of teachers’ assessment of AI using unstructured prompts and structured prompts in the evaluation of lesson plans enriched with the engineering design process

This hypothesis seeks to determine whether there is significant consistency between the assessment scores generated by AI systems using structured and unstructured prompts. The decision to distinguish between structured and unstructured prompts in this study is grounded in the Prompt Engineering Framework proposed by Zhou et al. (2022), which emphasizes that the format, specificity, and instructional clarity of prompts significantly affect the reliability and interpretability of outputs generated by large language models such as GPT-4 [87]. According to this framework, structured prompts provide the AI system with explicit task definitions, clear evaluation criteria, and performance expectations. This reduces the ambiguity of the input and narrows the model’s output space, enabling it to generate more focused, replicable, and valid responses. In contrast, unstructured prompts, characterized by vague or open-ended input, allow broader interpretation but increase the cognitive burden on the model. This can result in less predictable and less consistent outputs, as the model lacks specific guidance on what constitutes quality or adequacy in a given context. Therefore, employing both prompt types in this study serves a dual purpose:

1. Methodological Robustness – It allows a systematic comparison of how prompt structures influence AI evaluation quality, consistency, and alignment with expert judgments.
2. Theoretical Insight – It operationalizes the Prompt Engineering Framework to explore how AI prompt format impacts the validity of educational assessment, thus contributing to the ongoing discourse on the effective use of AI in education.

This differentiation is not only central to the internal validity of the study but also offers actionable insights for educators and instructional designers aiming to use AI responsibly in the development and evaluation of curriculum materials.

## Method

### 1. Research design

This study aims to evaluate lesson plans incorporating engineering design processes prepared by teachers, comparing the evaluations by experts and artificial intelligence, and testing the consistency between these evaluations. Given that both quantitative and qualitative data are utilized and support each other throughout the research process, an embedded mixed-methods design from Creswell’s (2014) typologies was adopted [88]. This design enhances the richness and depth of findings by increasing data diversity when addressing complex research problems [88]. Employing a mixed-methods design allows for a broader perspective in evaluating the results and enhances the validity of the research findings [89].

Quantitative data includes the total scores given by experts and artificial intelligence to the relevant lesson plans, while qualitative data encompasses the evaluation criteria and comments made by experts and artificial intelligence during the review of these lesson plans. For the collection of quantitative data, experts scored based on a pre-prepared criteria list, and the AI system performed scoring using both a structured and an unstructured prompt. These scores were analyzed to test the consistency between them quantitatively. Additionally, the internal consistency of the AI system was evaluated using structured and unstructured prompts.

Qualitative data includes elements such as the criteria used by AI and experts when evaluating lesson plans, the rationale for the scores given to themes in the criteria list, deficiencies, and suggestions. This data was addressed during the qualitative data collection process and was used to validate the findings from quantitative analyses and identify differences. Quantitative data provides an objective comparison of expert and AI scoring, while qualitative data details the evaluation criteria and comments behind these scores. This approach offers a comprehensive understanding of the evaluation processes and criteria, and the combined use of different data types provides multifaceted answers to research questions, thereby enhancing the accuracy and reliability of the results [90]. Moreover, the flexible nature of the mixed-methods design reveals nuanced differences between AI and human evaluations and plays a crucial role in understanding the implications of these differences for educational practice. In this context, choosing an embedded mixed-methods design enables in-depth access to both theoretical and practical aspects of the research, making it an ideal method for this study [88,89].

### 2. Study group

The participant group in this study consisted of 43 science teachers selected from five different provinces across Turkey, ensuring both regional and institutional diversity. These educators were involved in a nationally funded professional development initiative aimed at strengthening science instruction through the integration of Design-Based Learning (DBL) and educational technologies. Participants were selected using criterion sampling, a purposive method commonly used in qualitative and mixed-methods research to identify individuals who meet pre-established and theoretically relevant conditions [91]. This strategy ensures that the selected participants possess the specific characteristics necessary to meaningfully address the research objectives. Employing criterion sampling in this study was essential to align the teacher profiles with the pedagogical focus of DBL integration, thereby enhancing the internal validity and interpretability of findings.

The first key inclusion criterion was regional representation. A total of 43 teachers were selected from 12 provinces, each representing one of the seven official geographical regions of Turkey. This geographic spread was designed to ensure that the study captured a wide range of educational environments, regional practices, and socio-cultural dynamics. Regional representativeness is critical in innovation-focused educational research, as the adoption and implementation of pedagogical practices often vary significantly depending on local conditions [90]. Another essential selection criterion was the teachers’ participation in intensive professional development focused on DBL pedagogy. All participants completed 72 hours of in-person training and 25 hours of online instruction, followed by a four-month mentorship phase during which they developed, applied, and revised lesson plans. This extensive preparation ensured a standardized level of understanding and readiness among participants regarding DBL principles and technology integration. Another inclusion criterion was institutional and socio-economic diversity. Participants were drawn from Science and Art Centers (n = 6), general secondary schools (n = 33), and boarding schools in socioeconomically disadvantaged regions (n = 4). This diversity allowed the study to assess how DBL integration varies across different school types and community contexts. Research has shown that pedagogical innovations are not uniformly adopted across institutional settings; rather, their effectiveness and sustainability depend on contextual responsiveness [92].

The participant group also exhibits variation in academic qualifications. Twenty teachers hold only undergraduate degrees, while the remaining either hold or are pursuing advanced degrees: 13 hold master’s degrees, 6 are in the process of completing postgraduate education, 3 ware pursuing doctoral studies, and 1 holds a PhD. This educational heterogeneity enabled the study to explore potential differences in how teachers with varying academic preparation interpret and apply DBL principles. Prior research has demonstrated that higher academic qualifications often correlate with increased pedagogical content knowledge, especially in integrating technology into subject-specific instruction [93].

### 3. Research data and procedure

The data collected in this study includes the evaluation results of lesson plans developed by 43 science teachers over a four-month period. These teachers received training on integrating engineering design processes and lesson plans during a national mentorship project. Participants were recruited between October 10, 2022, and May 8, 2024. For the selection process, four teachers from each of the 12 different regions in Turkey were chosen, covering a diverse geographical area. These teachers were selected from those working in general and official regional boarding schools, as well as Science and Art Centers. A total of 52 lesson plans were analyzed by both experts and artificial intelligence software. The evaluation process followed three distinct methods. The workflow diagram below illustrates the steps taken during the study with the teachers and the production process of the analyzed lesson plans (Note: The program’s steps did not progress linearly; some steps were conducted simultaneously).

**Fig 2:**
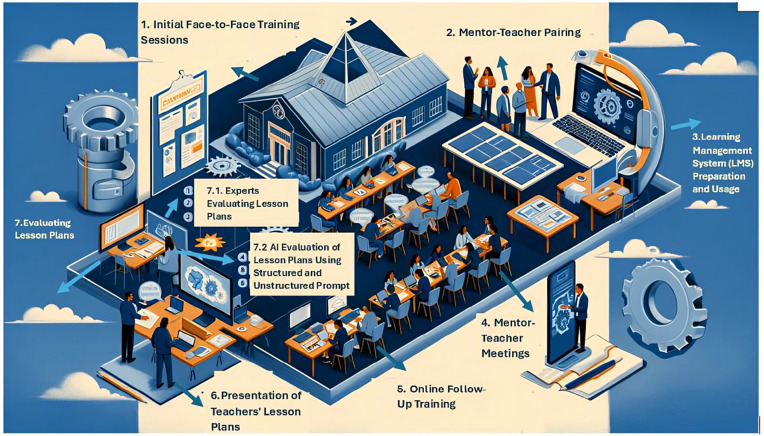
Research Process (Created by ChatGPT plugin diagram.ai).

As shown in Fig 2, the research was completed in seven steps. The details of these steps are provided below.

1. **Initial Face-to-Face Training (1 Week):** Within the scope of a national project, a one-week face-to-face practical training was given to a group of science teachers on integrating engineering design processes into their lesson plans. This training comprised introductory workshops and educational sessions aimed at providing a solid foundation for the teachers. During the training process, the goal was to enhance the teachers’ knowledge and skills in engineering design processes, technology integration, creativity, and lesson plan preparation.
2. **Mentor-Teacher Pairing (1 Week):** Following the training, expert mentors in engineering design processes and lesson plan preparation were assigned to support the teachers throughout the process. Mentors were carefully selected and paired based on their areas of expertise, teaching styles, and the needs of the teachers. This pairing process aimed to enable teachers to prepare and implement more effective lesson plans by receiving mentorship support.
3. **Learning Management System (LMS) Preparation and Use (4 Weeks):** During the process, an LMS was designed to support teachers’ applications in their own schools. All users were registered on this system, and resources and videos on engineering design processes, lesson plan preparation, and technology integration were provided through the system. Additionally, the LMS was used to facilitate communication between mentors and teachers, allowing continuous communication and collaboration throughout the process.
4. **Mentor-Teacher Meetings (8 Weeks):** Together with mentors, teachers developed lesson plans, implemented these plans in their classrooms, and conducted preliminary applications of the lesson plans. During the implementation process, mentors observed the lessons and provided constructive feedback to the teachers. Similarly, lesson plans were evaluated through the LMS, and feedback was provided to the teachers. In this process, the strengths and areas for improvement of the lesson plans were discussed, and the progress of the teachers was monitored.
5. **Online Follow-Up Training (1 Week):** Following all these processes, one-week synchronous lessons were conducted with the teachers through the LMS, covering topics such as experience sharing, technology integration, and creativity. The purpose of these synchronous lessons was to assess the teachers’ current status, address deficiencies, and support their professional development.
6. **Teachers’ Presentation of Lesson Plans (4 Weeks):** At the end of the training, teachers were asked to finalize their lesson plans incorporating the engineering design process and upload the resulting lesson plans to a cloud system for analysis. A total of 52 lesson plans were uploaded to the cloud system as part of this process. The lesson plans obtained from the relevant examinations were categorized for evaluation and prepared in a format suitable for assessment by both human experts and artificial intelligence. Teachers prepared their lesson plans within the framework of a lesson plan preparation template developed by the researchers. The lesson plan template is structured to include the five stages of the engineering design process. The sections of the lesson plan template and the instructions provided to the teachers are presented in theTable 1.

**Table 1:** Engineering design process criteria table.

Sections of the Lesson Plan	Sections of the Lesson Plan
Problem Identification	✔ The stage includes presenting the problem context, stating the problem situation, and defining the problem. ✔ The should design a problem situation with specific criteria and constraints that is suitable for generating multiple solutions and employing research-inquiry processes, grounded in real-life context [9,20–22]. ✔ The lesson plan should first introduce the problem context, then present the problem situation, providing students the opportunity to determine what is required in the problem.
Development of Possible Solutions	✔ The teacher should plan activities appropriate for a research-inquiry strategy to help students acquire the knowledge and skills needed to solve the problem (experiments, discussion, question-answer) [9,52]. ✔ Lesson plans should be designed to allow students to develop individual solutions to the problem and share them with their peers.
Selection of the Best Solution	✔ Steps should include students evaluating their proposed solutions within the context of the problem’s criteria and limitations, engaging in the optimization process known as in engineering, and choosing one to design and test the prototype [55]. ✔ Teachers can use techniques such as decision trees and decision matrices to help students evaluate solution proposals according to criteria and constraints. ✔ At this stage, students are expected to explain why the solution they chose is the best. Teachers should carefully use concepts like “trade-offs” in their explanations.
Prototype Creation and Testing	✔ Plans should be made to give groups the opportunity to create their solutions. Before implementing their chosen solutions, a plan should be made for the teachers to question group decisions. ✔ Teachers should explain how the proposed product/model/solution will be tested [94]. (For example, if a rubric is to be used, it should be shared with students from the problem identification stage.) Plans involving testing of students’ designs and improving their solutions after testing should be explained. ✔ Plans should be made for students to explain their improvements and retest them as needed.
Communication	Plans must be made for groups to present their designs and for evaluations to be conducted in the context of adherence to criteria [9].

Table 1 outlines the components and expectations of the engineering design process embedded in the lesson plan template. These criteria served as the basis for evaluating whether teachers effectively integrated problem-solving, creativity, iteration, and communication elements into their lesson designs.

**7.1. Experts Evaluating Lesson Plans:** A total of five expert evaluators were selected using purposive sampling based on disciplinary expertise and relevance to the engineering design pedagogy.

- Three experts were associate professors in science education, all with specialized research and practice in STEM education, engineering design processes, and teacher preparation. They were also part of the mentoring team in the national DBL project associated with this study.
- One expert held an associate professorship in Computer Education and Instructional Technology, with specialization in technology integration, instructional design, and STEM-focused teacher development.
- One expert was an associate professor in Educational Sciences, whose work focuses on pedagogy and the implementation of design-based learning in teacher education.

The disciplinary range and methodological experience of these experts provided a comprehensive perspective for evaluating lesson plans across multiple criteria. Their profiles ensured validity in assessing the pedagogical quality and fidelity of engineering design integration in the lesson plans.

Before the expert evaluation, a criteria list incorporating the engineering design process was prepared by experts. The preparation process for the criteria list was carried out in a detailed and careful manner to evaluate the engineering design process activity plans. First, the purpose and scope of the activities to be evaluated were determined. Then, the critical components of the engineering design process and the evaluation criteria for these components were identified. Different performance levels (e.g., expected level, acceptable, needs improvement) were defined for each criterion, and each level was supported by detailed explanations. Accordingly, it was decided that the criteria list would include 13 sub-items covering the engineering design process: Problem Identification, Science/Mathematics Achievements, Clarity, Student Context, Multiple Solutions, Research-Inquiry, Testability, Other STEM Disciplines, Development of Possible Solutions, Selection of the Most Appropriate Solution, Prototyping and Testing, and Communication. For each item, three performance levels were defined: “Expected Level,” “Acceptable,” and “Needs Improvement.” The validity of the criteria list and prompt used in this study was ensured in several steps. The criteria list prepared for evaluating the lesson plans incorporating engineering design processes was developed together with subject matter experts and teachers to ensure content validity. Content validity assesses whether the measurement tool fully covers the subject [95]. In preparing the rubric, the critical components of the engineering design processes and the evaluation criteria for these components were identified, different performance levels were defined for each criterion, and the criteria list was included with the approval of experts.

Face validity refers to the extent to which a measurement tool, such as a rubric, appears to measure the intended construct based on expert and user judgment. In this study, face validity was ensured by determining whether the criteria in the criteria list were consistent with the structures mentioned in the literature related to the engineering design process and teaching practices. The criteria list was based on criteria overlapping with the engineering design processes suggested in the literature. The criteria list was tested through pilot applications. In the preparation process of such lists, validity and reliability analyses are generally ensured through expert opinions [96]. Moskal and Leydens (2000) state that the reliability of criteria lists can be increased by ensuring consistency among different evaluators [95]. In this context, the criteria list was reviewed by expert teachers and academics in the field and revised according to their feedback. The applicability and comprehensibility of the items were tested through pilot applications, and issues encountered during these tests were identified. Based on the feedback, the criteria list was revised and finalized. The maximum score obtainable from the criteria list is 36, and the minimum score is 12. This process aims to support teachers in preparing more structured and goal-oriented lesson plans and to help students succeed in the engineering design processes.

Based on the 13-item criteria list obtained during the research process, experts evaluated the lesson plans available in the cloud system. In this process, a total of 52 lesson plans were individually scored for each criterion, and the evaluation justifications were detailed. The scores given for each criterion were summed to determine the overall quality of the lesson plans and the level of integration of the engineering design processes. Experts provided detailed explanations for the scores given for each criterion, highlighting the strengths and weaknesses of the lesson plans. To ensure the reliability of expert ratings, we conducted an inter-rater reliability analysis among the five experts using the intra-class correlation coefficient (ICC). The results indicated excellent consistency (ICC = 0.81, p < .001), suggesting that the experts’ evaluations were highly aligned. According to established benchmarks [96,97], ICC values above 0.81 are considered “good,” supporting the rationale for using expert evaluation as the reference standard in subsequent comparisons with AI-based assessments. All evaluation results, including the scores in each category and the justifications for these scores, were recorded in an electronic spreadsheet software. An example of an expert evaluation is provided Annex 1: Lesson plan 5 from the expert evaluation.

**7.2. AI Evaluation of Lesson Plans Using Structured and Unstructured Prompts:** Prompts play a vital role in guiding artificial intelligence (AI) systems to produce accurate and relevant outputs. By optimizing the text provided to AI models, prompts ensure correct interpretation and generation of pertinent, detailed results [98]. Both structured and unstructured prompts are crucial for AI systems to generate effective outputs and complete tasks. Structured prompts are designed to include elements such as clarity, precision, contextual information, desired format, and level of detail, optimizing the information processing of AI models. This allows AI models to break down information into manageable blocks, contextualize requests, and and generate responses in the required format and level of detail [99]. On the other hand, unstructured prompts do not follow a predefined format and rely on AI models to interpret the provided input and produce results based on that input [100]. Both structured and unstructured prompts are necessary for AI systems to generate effective outputs.

In this context, comparing the evaluation scores produced by structured and unstructured prompts constitutes one of the sub-goals of this study. For this purpose, two types of prompts were prepared, and the evaluation results of the lesson plans were conducted using these two different evaluation methods. The design process of both structured and unstructured prompts was carried out through interdisciplinary expert collaboration. Specifically, the team included one associate professor specializing in Computer and Instructional Technologies, three associate professors in Science Education, and one experienced educator with field-based instructional experience. The prompts were constructed based on a validated and reliable 13-item expert evaluation rubric grounded in the components of the engineering design process. Once the initial versions were prepared, they were reviewed by an external reviewer—an associate professor in Computer and Instructional Technologies—to ensure consistency, clarity, and methodological soundness. Revisions were made in response to the reviewer’s feedback. Furthermore, preliminary trials were conducted with the developed prompt versions, and the prompts were refined based on the evaluation outcomes to improve usability and alignment with the evaluation framework. The lesson plans were evaluated using ChatGPT 4.5, the subsequent version of the GPT-4-based large language model developed by OpenAI. Access to the model was provided through the ChatGPT Plus web interface, and it was selected for its advanced natural language processing capabilities and high level of accuracy. Each lesson plan was individually uploaded into the ChatGPT 4.5 environment using the “attach” feature and assessed through both structured and unstructured prompts. The input prompt length was limited to 3,000 characters, and the model output was restricted to approximately 1,000 words. To balance response diversity with output consistency, the temperature setting was configured at 0.7. To support reproducibility, all technical parameters—including the prompt templates and evaluation format—were described in detail. Example prompt templates used in the study are presented within the Method section. Data processing was initially conducted using Microsoft Excel, while all statistical analyses were carried out using the Jamovi statistical software.

The first prompt used in the study, the structured prompts, were designed to include elements such as clarity, precision, contextual information, desired format, and level of detail control during the evaluation process. The unstructured prompts, on the other hand, did not follow a predefined format and were structured to allow ChatGPT to interpret the provided input and produce results based on that input without much detail. Details and example evaluation results related to both the structured and unstructured prompts are provided below:

***Unstructured Prompt Evaluation:*** A total of 52 lesson plans were evaluated using the unstructured prompt, and the results were listed along with their justifications. Examples of evaluations conducted using the unstructured prompts are presented below. These examples demonstrate the evaluation of lesson plans using unstructured prompts and compare the resulting outcomes with those from structured prompts. For comparison and understanding the differences, Lesson Plan 5 from the Unstructured Prompt Evaluation is provided as in Annex 2- Unstructured Prompt Ai Evaluation Results: Lesson Plan 5.

***Structured Prompt Evaluation:*** In the third and final step, a structured prompt was used. The structured prompt was developed based on the evaluation criteria used by the experts. Additionally, expert opinions were gathered before the prompt was employed. You can refer to Annex 2: Structured Prompt Used for Evaluating Lesson Plans for the sample prompt used. To clearly compare the differences, the evaluation results of Lesson Plan 5, which was previously given as an example and evaluated using the structured prompt, can be examined in detail in Annex 2: Structured Prompt AI Evaluation Results.

A total of 52 lesson plans were scored along with their justifications based on expert evaluations, structured, and unstructured prompt assessments. The obtained scores were uploaded into an analysis program to be analyzed as quantitative data.

Qualitative data included the criteria used by AI and experts when evaluating the lesson plans, the justifications for the scores given to the themes in the criteria list, deficiencies, and suggestions. These data were addressed during the qualitative data collection process and were used to validate the findings obtained from quantitative analyses and identify differences. Quantitative data provides an objective comparison of the scores given by experts and AI, while qualitative data details the evaluation criteria and comments behind these scores. This approach ensures a comprehensive understanding of the evaluation processes and criteria. The combined use of different data types provides multifaceted answers to the research questions, thereby enhancing the accuracy and reliability of the results [96]. Moreover, the flexible nature of the mixed-methods design reveals nuanced differences between AI and human evaluations and plays a crucial role in understanding the implications of these differences for educational practice. In this context, choosing an embedded mixed-methods design enables in-depth access to both theoretical and practical aspects of the research, making it an ideal method for this study [88,89].

### 4. Data analysis

In this study, the evaluation scores of lesson plans incorporating the engineering design process, prepared by teachers, were compared using Bland-Altman and Intraclass Correlation Coefficient (ICC) analyses. These evaluations included scores from expert evaluations, structured prompts, and unstructured prompts using artificial intelligence. The Intraclass Correlation Coefficient (ICC) was selected to evaluate the level of agreement between expert and AI-generated scores because it is particularly suitable for assessing consistency among raters when the measurement scale is continuous and multiple evaluators are used [101]. In contrast to other correlation metrics like Pearson’s r, which only measures linear association, ICC captures both consistency and absolute agreement across raters. Given that this study involves comparing structured and unstructured AI prompt scores with expert evaluations on lesson plans, ICC provides a robust framework to assess inter-rater reliability and the stability of these scoring approaches.

The Bland-Altman analysis is a widely used method to evaluate the agreement between two measurement methods. In this study, it was used to examine the differences and directions between the expert evaluations and AI evaluations of the lesson plans prepared by teachers. This analysis provides a visual representation of the agreement by calculating the mean difference (bias) between the two measurements and the confidence intervals of this difference (limits of agreement) [102]. The Bland-Altman analysis is considered robust as it allows for a detailed examination of individual differences between the two measurement methods [103]. Since the Bland-Altman analysis assumes that the measurement differences are normally distributed, the data set to be analyzed is expected to show a normal distribution.

While ICC indicates the overall statistical consistency between measurements, it does not reveal whether one method systematically tends to score higher or lower than the other. Therefore, using Bland-Altman analysis alongside ICC enables a more comprehensive examination of evaluation reliability by assessing not only the degree of correlation but also potential directional biases and the limits of agreement between scoring methods. This multidimensional approach is particularly critical in fields like education, where precision and fairness in assessment are essential.

In this study, the Shapiro–Wilk test was employed to assess the normality assumption of three distinct evaluation score sets: expert evaluations, structured prompt AI evaluations, and unstructured prompt AI evaluations. The results indicated that all three distributions were consistent with normality: Structured Prompt AI Scores (W = 0.915, p = 0.08), Expert Evaluation Scores (W = 0.960, p = 0.076), and Unstructured Prompt AI Scores (W = 0.978, p = 0.437). Given that all p-values exceeded the commonly accepted threshold of 0.05, the null hypothesis of normality was not rejected. The Shapiro–Wilk test is widely regarded as one of the most powerful normality tests, particularly suitable for small to moderate sample sizes [104]. A p-value greater than 0.05 typically suggests that the data are not significantly different from a normal distribution, allowing researchers to proceed with parametric statistical methods such as the Bland–Altman agreement test. The validity of Bland–Altman analysis further requires the compared measures to be independent and approximately normally distributed [103]. In the present study, these assumptions are met, as the datasets were derived from independent evaluations by human experts and two distinct AI prompting conditions.

The intraclass correlation coefficient (ICC) is a method used to assess the reliability and consistency of measurements within a group. In this study, ICC was used to evaluate the consistency of the lesson plan scores given by different evaluators (experts and artificial intelligence). ICC determines the degree of agreement by calculating the variance among evaluators relative to the total variance [105]. ICC quantitatively expresses the agreement among evaluators, thus highlighting the reliability of the scoring processes [101], making it a robust analysis. ICC analysis assumes that the measurements are normally distributed, which, as previously mentioned, is the case for the data set in this study. Additionally, like Bland-Altman, ICC assumes that the measurements are independent. The three different sets of scores in this study can be considered independent.

The 13-item criteria list used in the research was the basis for evaluating the lesson plans on the cloud system by experts. In this process, a total of 52 lesson plans were individually scored for each criterion, and the evaluation justifications were detailed. Both experts and the AI system provided detailed justifications for the scores given for each criterion, highlighting the strengths and weaknesses of the lesson plans. These justifications were used to explain the underlying reasons for the results obtained from the quantitative data. For the first hypothesis, the highest and lowest differences among the evaluations were compared using the justifications from the expert, structured, and unstructured prompt AI evaluations. For the second and third hypotheses, three evaluations were compared to analyze similarities and differences in the justifications. The data were analyzed using content analysis, coded in two cycles. In the first phase, open coding was used to break down, examine, compare, conceptualize, and categorize the data [105]. This process is crucial for developing initial categories and concepts from raw data. In the second phase, deductive coding was used to reorganize the results of the open coding according to a pre-established and literature-supported criteria list [106]. This criteria list was developed by experts to evaluate the integration of engineering design processes into lesson plans. The results revealed the underlying reasons for the differences in the various evaluation outcomes.

### 5. Reliability and validity of the study

Various steps were taken to ensure the reliability and validity of the study. Firstly, a mixed-methods design was used to collect both qualitative and quantitative data, allowing for a detailed examination of the data. The use of a mixed-methods design enabled a more comprehensive and in-depth analysis of the research findings [107]. Literature reviews and expert consultations were conducted in the development of the prompts. This process ensured that the prompts were developed based on scientific foundations, contributing significantly to their validity. Additionally, literature reviews and validity and reliability steps were followed in the creation of the criteria list. Content and construct validity were taken into account when determining the criteria [108].

Detailed descriptions were provided in the text to enrich the data and make the findings more meaningful. Appropriate and robust data analysis methods were chosen, including the use of ICC (Intraclass Correlation Coefficient) and Bland-Altman analysis, and their assumptions were tested. The use of these methods increased the accuracy and reliability of the data [102]. In the analysis of the qualitative data, support was obtained from experts, and credibility was ensured through direct quotations. This process made the qualitative data more valid and reliable and allowed for an accurate reflection of the participants’ views. Triangulation, a method used to increase validity in qualitative research, reinforced the accuracy of the findings by providing data diversity [109].

The involvement of experts at every stage of the research was another crucial factor that enhanced the scientific validity and reliability of the study. In this study, expert opinions were sought during the development of the criteria list and the AI evaluation systems, and necessary adjustments were made based on these opinions. The contributions of experts ensured that the measurement tools were developed based on scientific foundations, thereby enhancing the validity and reliability of the study. The results were presented and discussed in conjunction with the existing literature. This is important for evaluating whether the findings are consistent with the existing knowledge in the literature and for highlighting the contributions of the research [108].

### 6. Researchers’ roles and ethical considerations

While conducting the research, the researchers took a series of measures to ensure ethical standards. The first of these measures was obtaining approval from a university human research ethics committee. Additionally, since the research was conducted with teachers, permission was obtained from the Ministry of National Education. Participant teachers were clearly informed of their right to withdraw at any stage. In this research, the researchers played two different roles. In the project, they assumed an emic role, being more insiders than outsiders, as this research was part of a larger project where the researchers worked as instructors during the activities. However, they took on an etic role, being more outsiders, when collecting the data. The researchers did not interfere with the teachers’ preparation of their lesson plans. Feedback on the plans was given solely by their mentors, who also did not make any changes to the plans. The lesson plans submitted by the teachers were used without any alterations. Finally, the identities of the teachers were never disclosed to any third parties.

## Findings

The primary aim of this research is to examine the differences between AI systems and human evaluations of lesson plans developed by teachers trained in the engineering design process. It also seeks to assess the consistency between the two sets of evaluation scores. The results obtained are detailed below in alignment with the sub-objectives of the study.

### H1: The consistency between structured prompt AI evaluation scores and expert evaluation scores in assessing lesson plans enriched with the engineering design process is significant

The first comparison conducted in the study was between expert evaluation scores and structured prompt AI evaluation scores. To measure the reliability and degree of agreement between evaluators, the data were subjected to Intraclass Correlation Coefficient (ICC) analysis. The Oneway model and Single unit were used as the ICC model. The results obtained are presented in Table 2.

**Table 2:** Intraclass correlation coefficient results.

	Subjects	Raters	Subject variance		Rater variance	Residual variance	Consistency	ICC	P	f
Value	52	2	28.9	0.708	0.0999	11.8	0.710	0.708	0.000	5.85

According to the ICC analysis presented in Table 2, there is a 70.8% agreement among the evaluators in scoring the lesson plans prepared by teachers (ICC = 0.708). The F-test indicates that this level of agreement is statistically significant (F = 5.85, df1 = 51, df2 = 52, p = 0.000). The 95% confidence interval for the ICC ranges from 0.543 to 0.821, supporting a high degree of agreement among evaluators. This finding supports the H1 hypothesis. The evaluator variance, which represents the variance of scoring differences among different evaluators, is found to be 0.0999. This low value indicates that the evaluators generally give similar scores. The unexplained variance, however, is 11.8. This value suggests that some inconsistencies and variance still exist.

**Fig 3:**
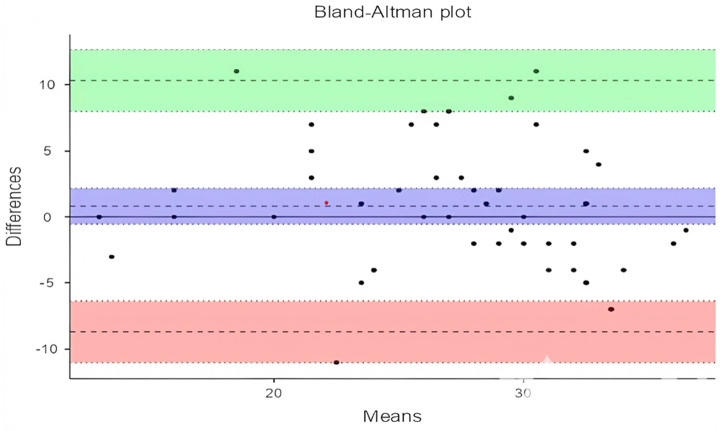
Bland-altman plot for sub-objective 1.

Although the ICC analysis shows a high level of agreement among evaluators in scoring lesson plans enriched with the engineering design process, the residual variance of 11.8 indicates that some inconsistencies between the two evaluators persist. To identify the source and direction of this discrepancy, a Bland-Altman analysis was conducted, and the results are presented in Table 3 and Fig 3.

**Table 3:** Bland-altman results for sub-objective 1.

	95% Confidence Interval		
	Estimate	Lower	Upper
Bias (n = 52)	0.808	−0.543	2.16
Lower limit of agreement	−8.700	−11.023	−6.38
Upper limit of agreement	10.315	7.993	12.64

The Bland-Altman analysis (Table 3) indicates that the mean difference between the two evaluators’ scores is 0.808 (95% CI: −0.543, 2.16). The lower and upper limits of agreement are −8.700 (95% CI: −11.023, −6.38) and 10.315 (95% CI: 7.993, 12.64), respectively. These findings suggest that there is generally a low difference between the evaluators’ scores, and the scoring is overall consistent. The horizontal line in the plot represents the mean difference (bias) between the two evaluators. According to the plot (Fig 3), the mean difference is approximately 0, indicating that the two evaluators generally provided similar scores. The upper (green) and lower (red) limits in the plot represent the 95% confidence intervals of the differences. The majority of the score differences fall within this range, indicating high consistency between the evaluators. The black dots in the plot represent the score differences for each lesson plan between the two evaluators. Most of these points are clustered around the mean difference line, with very few points falling outside the confidence intervals. This suggests that there is generally high agreement between the evaluators and that significant deviations are rare. There is no clear trend or systematic bias observed in the plot, indicating that the score differences are distributed independently of the average score.

Although the consistency between the two sets of evaluation scores is high, some notable differences are evident. To identify the reasons for these differences, the detailed justifications for the two lesson plans with the highest score discrepancies between expert evaluations and structured AI prompts were compared. These justifications include the reasons for the scores given by both AI and experts across the 13 criteria. The lesson plan with the highest score discrepancy (11 points) is Lesson Plan 5. The expert evaluation notes that the science and mathematics “achievements are only partially met and should be expanded to cover all achievements comprehensively.” In contrast, the AI evaluation states that the “science and mathematics achievements are fully met.” This discrepancy indicates that the AI evaluated the achievements more positively and sufficiently, whereas the experts adopted a more critical approach. There are also differences in the clarity of criteria and constraints. Experts emphasize that these areas are “not clearly presented to students and need more clarity” (1/3), whereas AI states that these areas are “clearly and effectively defined” (3/3). This indicates that AI made a more optimistic assessment regarding the identification and understanding of the problem. Regarding testability, experts find the lesson plan lacking, with uncertainties about “what and how students should test” (1/3). On the other hand, AI argues that the “testability process is detailed and sufficient” (3/3). This difference suggests that AI’s evaluation has a more holistic perspective, while experts focus more on details and shortcomings. Similar differences exist in the integration of other STEM disciplines. The expert evaluation notes that this integration is “limited and could be improved” (2/3), while AI finds this integration sufficient (3/3). This shows that AI considers interdisciplinary connections adequate, while experts expect broader integration. In the prototyping and testing processes, experts mention “uncertainties and lack of clarity” (1/3), while AI states that these processes are “clearly and practically defined” (3/3). Additionally, in terms of communication opportunities, experts emphasize that “students are not provided with enough opportunities to present and discuss their designs” (1/3), whereas AI finds that sufficient opportunities are provided (3/3). These differences show that AI tends to find the existing state sufficient and effective, while experts take a more critical and developmental approach. Generally, expert evaluation highlights the deficiencies and areas for improvement in the lesson plan, whereas AI evaluation views the current state more positively and adequately.

In Lesson Plan 14, a 12-point difference between the expert evaluation and the structured prompt AI evaluation was observed. The expert evaluation criticizes the lesson plan as follows: “The activity is not planned to include the steps of design-based learning. A very general real-life problem is presented, and parts related to evaluating the success of the design are included, but the problem is not suitable for this.” This statement emphasizes that the lesson plan lacks sufficient depth and that the presented problem is not aligned with the learning steps. In contrast, the AI evaluation assesses the lesson plan more positively: “The problem identification has been comprehensively done, and the necessary qualities and obstacles are clearly defined.” This indicates that the AI considers the problem definition and the presented constraints to be clear. Furthermore, the experts gave a score of “Science/Mathematics Achievements: 1,” indicating that the science and mathematics learning objectives were not adequately met, while the AI stated, “The learning achievements are clearly stated, and the necessary materials and processes for evaluation have been planned,” awarding a score of 3/3. This suggests that the AI believes the science and mathematics learning objectives have been effectively addressed. In the expert evaluation, a score of “Research-Inquiry: 1” was given, indicating inadequacy in this area, while the AI mentioned, “The lesson plan supports research and inquiry, but these processes could be further detailed,” and awarded a score of 2/3, acknowledging the need for potential improvement. Lastly, regarding the prototyping and testing process, experts criticized it by saying “only appropriate guidelines are included in the presentation phase,” while the AI stated, “The testing and evaluation of the prototype have been planned, but the implementation details are missing,” rating this process as acceptable with a score of 2/3.

Overall, the expert evaluations exhibit a more critical and detailed approach, highlighting deficiencies and areas for improvement. In contrast, AI evaluations generally find the current state adequate and offer fewer detailed suggestions for improvement. Experts identify shortcomings and provide improvement suggestions, while AI evaluates the current state and emphasizes general acceptability. The variance in scores may be attributed to this difference in perspective between the two evaluators.

### H2: The consistency between unstructured prompt AI evaluation scores and expert evaluation scores in assessing lesson plans enriched with the engineering design process is significant

To measure the reliability and degree of agreement between the unstructured prompt AI evaluation scores and expert evaluation scores for the second sub-objective, the data were subjected to ICC analysis. The Oneway model and Single unit were used for the ICC model. The results are presented in Table 4.

**Table 4:** ICC results for sub-objective 2.

Subjects	Raters	Subject Variance	Rater Variance	Residual Variance	Consistency	Agreement	ICC	F	df2	p
52	2	4.154	3.695	24.229	0.146	0.130	0.076	1.165	52	0.292

According to the ICC analysis presented in Table 4, there is a 7.6% agreement among the evaluators in scoring the lesson plans prepared by teachers (ICC = 0.076). The F-test indicates that this level of agreement is not statistically significant (F = 1.165, df1 = 51, df2 = 52, p = 0.292). This finding rejects the H2 hypothesis. The 95% confidence interval for the ICC ranges from −0.197 to 0.339, supporting a low degree of agreement among evaluators. The evaluator variance, which represents the variance of scoring differences among different evaluators, was found to be 3.695. This low value indicates that the evaluators rarely gave similar scores. The unexplained variance, however, was calculated to be 24.229. This suggests significant inconsistencies and variance in the scores.

**Fig 4:**
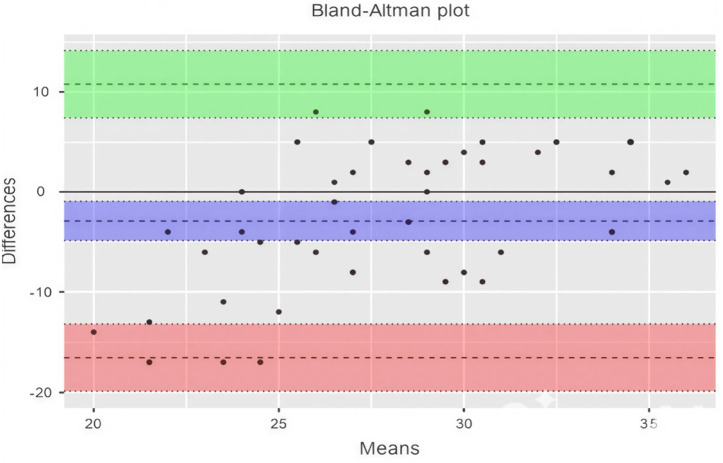
Bland-altman plot for sub-objective 2.

Although the ICC analysis shows low agreement among evaluators in scoring lesson plans enriched with the engineering design process, the residual variance of 24.229 indicates substantial discrepancies between the two evaluators. To identify the source and direction of this discrepancy, a Bland-Altman analysis was conducted, and the results are presented in Table 5 and Fig 4.

**Table 5:** Bland-altman results for sub-objective 2.

	95% Confidence Interval		
	Estimate	Lower	Upper
Bias (n = 52)	−2.885	−4.823	−0.947
Lower limit of agreement	−16.528	-19.862	-13.195
Upper limit of agreement	10.759	7.426	14.092

When examining Table 5, it is observed that the systematic bias between the two evaluators is −2.885. This indicates that the unstructured prompts tend to give scores approximately 2.885 units higher on average than the expert evaluations. The 95% confidence interval for this bias ranges from −4.823 to −0.947, confirming a systematic underestimation. Additionally, the wide limits of agreement, ranging from −16.528 to 10.759, reveal significant variability in the differences between the two methods. This wide range indicates that unstructured prompts consistently give higher scores compared to expert evaluations, and these differences exhibit substantial variability. The presence of points (Fig 4) near or even exceeding the limits on the graph further highlights the issues of agreement and consistency between the two methods. These findings raise concerns about the reliability and interchangeability of unstructured prompts with expert evaluations, especially in situations requiring high accuracy and consistency.

To determine the reasons for the discrepancy, the evaluations of the three lesson plans with the highest score differences were compared. One of these, Lesson Plan 13, shows a 17-point difference between expert and AI evaluations. The expert evaluation notes that the lesson plan does not include the steps of design-based learning and is based on a very general real-life problem.

*Expert Evaluation Excerpts: “The activity is not planned to include the steps of design-based learning. A very general real-life problem is presented, and parts related to evaluating the success of the design are included, but the problem is not suitable for this.”*

Experts gave low scores to the lesson plan across all criteria, including constraints, science/mathematics achievements, clarity, student context, multiple solutions, research-inquiry, testability, other STEM disciplines, development of possible solutions, selection of the most appropriate solution, prototyping and testing, and communication. Experts stated that “the activity should be planned to include a design problem,” indicating that this aspect is lacking. In contrast, the AI evaluation was more positive about the lesson plan. The AI evaluation found the problem identification acceptable with the statement “the problem definition is interesting and linked to education.” Similarly, for constraints and criteria, the AI noted that “more information is needed on how students will assess and apply the criteria,” but still rated it as acceptable. This indicates that the AI considers the structures supporting student interactions and learning processes based on the existing information sufficient but acknowledges that adding more details would be beneficial. For the clarity criterion, the AI explained that “the design problem and achievements are generally well-expressed,” rating it as expected level. Likewise, the AI positively evaluated the student context as “linked to students’ lives and well-addressed in the social context.” This indicates that the AI evaluates the integration of learning materials learning materials with students’ real-world experiences and social environments as an important measure. However, the AI also gave low scores in areas like testability and other STEM disciplines, citing issues such as the lack of testing processes and limitations to science subjects. Overall, the AI evaluation gave higher scores, but still highlighted some shortcomings. While experts focused on the deficiencies of the lesson plan and noted that significant improvements were needed, the AI found certain areas sufficient. The most significant difference was in the definition of constraints and criteria; experts found these areas lacking, while the AI considered them adequate.

“Another difference noted in Lesson Plan 19 (scored 17 points), according to expert evaluation, is that the activity does not include design-based learning steps and focuses on a very general everyday life problem. The design problem should be structured based on explicit criteria and constraints. The plan does not sufficiently support science and math learning outcomes, lacks a clear and structured format, fails to consider the student context, and does not include multiple solution pathways. Research and inquiry steps are missing, testability has not been ensured, and other STEM disciplines are not adequately represented. The development of potential solutions, selection of the most suitable solution, prototype creation, testing processes, and communication skills are also found to be deficient.

*Quotes from Expert Evaluation: ‘The activity was not planned to include design-based learning steps. A very general everyday life problem was presented. It should be structured around a design problem with criteria and constraints.’*

The artificial intelligence evaluation, however, has been found more positive in some criteria. AI notes that the lesson plan ‘focuses on a real-world problem such as seed scarcity resulting from climate change,’ enabling students to define the problem and establish real-life connections. ‘According to AI, the cornerstones of the research and idea generation process are giving students the opportunity to brainstorm to identify the needs related to the problem and encouraging them to gather information from various sources. In the prototyping phase, ‘students monitoring the germination processes using fruit seeds and tracking this process with graphs, as well as testing and improving the prototypes of the solutions created,’ has been deemed sufficient. In the solution evaluation and communication phase, AI approves of the section where ‘...solution proposals are discussed within the group, and techniques like decision matrices are used to select the most suitable solution.’ Lastly, AI contends that this lesson plan ‘... comprehensively addresses the basic components of the engineering design process and includes activities that facilitate students’ understanding of this process,’ and states that it is justified in receiving a high score.

“Another lesson plan worth reviewing, Lesson Plan 29, exhibits a 17-point discrepancy between two evaluators. This significant difference highlights the subjective variations in interpreting and applying evaluation criteria, and also provides insights into which elements are emphasized in the evaluation of lesson plans. The expert has noted that the lesson plan should ‘include measurable criteria and constraints,’ emphasizing that measurements must be based on objective and concrete data. The expert’s partial approval of the lesson plan regarding science and mathematics achievements stems from the activity not fully encompassing the targeted science outcomes. This indicates the expert’s expectation that instructional materials align completely with educational objectives.. Experts positively viewed the inclusion of ‘a step-by-step model design for students,’ yet, despite this positive aspect, they highlighted deficiencies in other stages of the design process. This critique underscores the importance of comprehensively addressing the entire process, not just one phase. Particularly, the statement, ‘However, it needs to be revised from the beginning with a problem that allows for the development of multiple solutions and is planned according to the stages of the design process,’ suggests the need for diversity and flexibility in the design process to foster students’ creative thinking skills. The artificial intelligence analysis states that the lesson plan ‘provides students with real-world problems, facilitating active learning opportunities to understand the engineering design process,’ and ‘offers students the chance to experience engineering processes such as problem identification, solution development, and prototyping through a trifle-making activity.’ Additionally, it is mentioned that the plan ‘successfully integrates science and mathematics, offering students an interdisciplinary learning experience by correlating respiratory system and health topics with mathematical calculations.’ According to the AI evaluation, such connections ‘allow students to understand the relationships between different disciplines and apply their knowledge through a holistic approach.’ The lesson plan, according to AI, ‘allows students to assess their own work through analytical rubrics,’ and ‘provides feedback for students to reflect on their learning and continuously improve.’ Overall, contrary to the expert evaluation, the AI states that this lesson plan ‘effectively encompasses engineering and design processes, encourages active student participation, and enriches learning by establishing interdisciplinary connections’.

The source of these discrepancies may lie in the expert evaluation’s more comprehensive educational design perspective, focusing on deficiencies in the lesson plan and assessing it within a framework of specific pedagogical standards. On the other hand, artificial intelligence may process the given information at a superficial level, highlighting some positive elements more prominently. This suggests that AI can sometimes lack a broader perspective and may be limited in understanding specific educational standards or in-depth pedagogical assessments. Consequently, while expert evaluations provide a more detailed examination of the lesson plan’s content richness, AI highlights certain positive aspects but remains limited in conducting a holistic educational design assessment.

### Hypothesis 3: The consistency between AI evaluation scores obtained using unstructured prompts and structured prompts in assessing lesson plans enriched with the engineering design process is significant

In the final sub-objective of the study, the differences between AI evaluation scores obtained using structured and unstructured prompts were investigated, and an Intraclass Correlation Coefficient (ICC) analysis was conducted to measure reliability and determine the degree of alignment. The results are presented in Table 6.

**Table 6:** ICC results for sub-objective 3.

	Subjects	Raters	Subject variance	Rater variance	Residual variance	Consistency	Agreement
Value	52	2	2.599	1.692	22.106	0.105	0.098

**Fig 5:**
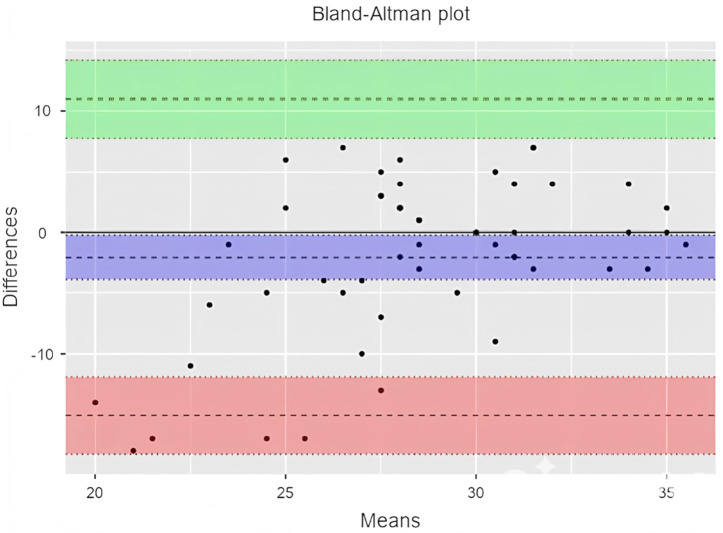
Bland-altman plot for sub-objective 2.

When Table 6 is examined, it reveals that there is a 6.9% agreement among evaluators in scoring lesson plans prepared by teachers (ICC = 0.069). According to the F test, this level of agreement is not statistically significant (F = 1.147, df1 = 51, df2 = 52, p = 0.312), indicating the rejection of Hypothesis 1. The 95% confidence interval for the ICC ranges from −0.204 to 0.332, supporting a low degree of agreement among evaluators. The evaluator variance, which represents the variance in scoring differences among different evaluators, is determined to be 1.692 for this study. This value indicates that evaluators typically assign different scores. The ICC analysis demonstrates that there is low agreement among evaluators when scoring lesson plans enriched with the engineering design process, and the residual variance of 22.106 suggests the presence of some inconsistencies. To determine the source and direction of this difference, a Bland-Altman analysis was conducted, with results presented in Table 7 and Fig 5.

**Table 7:** Bland-altman results for sub-objective 3.

	95% Confidence Interval		
	Estimate	Lower	Upper
Bias (n = 52)	−2.058	−3.909	−0.207
Lower limit of agreement	−15.090	−18.274	−11.906
Upper limit of agreement	10.975	7.791	14.159

$Upon examining Table 7, a systematic bias of −2.058 indicates that the evaluation scores using structured prompts are, on average, 2.058 units lower than those using unstructured prompts. The 95% confidence interval for this bias, ranging from −3.909 to −0.207, signifies that the deviation is statistically significant. Additionally, the wide limits of agreement, ranging from −15.090 to 10.975, highlight significant variability between the two methods. This variability causes some points to approach or exceed these limits of agreement. Despite the first method consistently underestimating the values, the degree of differences between measurements shows considerable variability. An examination of Fig 5 reveals that although most observations fall within the acceptable limits, several values approach or even exceed the lower and upper limits of agreement. This pattern underscores the inconsistency in measurement differences between the two methods and further supports the conclusion that they may not be reliably interchangeable. These findings suggest that substituting one method for the other could result in issues of precision and reliability.

The evaluation conducted using both structured and unstructured prompts by artificial intelligence has revealed a lack of consistency in the assessment scores. Additionally, findings that support this data can also be observed in the detailed criteria for evaluating lesson plans. In this context, the first three lesson plans that showed the most significant differences following evaluations with structured and unstructured prompts have been compared. In Lesson Plan 51, the difference between the unstructured prompt AI evaluation and the structured prompt AI evaluation scores was determined to be 17 points. Based on the structured prompt AI evaluation results, the lesson plan explicitly aims to utilize renewable energy sources and establish systems to replace fossil fuels, thereby earning a score of 3 for the ‘Problem Identification’ criterion. However, in the ‘Testability’ criterion, only 1 point was awarded because “the performance of the established system needs to be assessed, but this process is not sufficiently detailed in the lesson plan.” It has been noted that the plan successfully integrates different STEM disciplines; however, it only received 2 points for communication because “additional explanations are needed to better illustrate to students how interdisciplinary interactions work.”. During the solution development process, the plan was noted to “be more strategic and directive.” It was mentioned that “support should be provided to students in the process of selecting among various design alternatives,” for which 2 points were awarded. For the process of selecting the most appropriate solution to operate more effectively, it was stated that different solution paths evaluation criteria should be provided to students, receiving only 1 point, indicating that this process was inadequate. In the prototype creation and testing process, it was noted that “it needs to be more detailed to reinforce students’ engineering skills,” and “a clear guide on how the test results should be evaluated is required.” In the communication area, although the student project presentation processes are well-organized, it was emphasized that “feedback mechanisms that further enhance presentation skills need to be added.” The structured prompt evaluations detail each criterion, while the unstructured prompt AI evaluations tend to assess more generally. The AI evaluation conducted with an unstructured prompt indicates that the project “aims to demonstrate how energy production can be achieved using wind and solar energy,” highlighting the clarity of the problem from this perspective. Additionally, it “emphasizes the necessity of using renewable sources instead of fossil fuels alongside the production of energy through environmentally friendly methods,” thus providing a clearer explanation of the process. According to AI, the project “encourages the active use of its potential in terms of wind and solar energy” and includes feasible and most appropriate solutions by “teaching students how these resources can be integrated with the increasing energy demand.” It also addresses “minimizing harm to nature while meeting energy needs” and “informing students about sustainable living practices,” which can be related to the student context. Among the materials used are “wind turbine and solar panel assembly kits,” which “provide students with practical application opportunities,” thereby meeting the criteria for Prototype Creation and Testing. However, in the unstructured prompt, there is no assessment regarding the integration of STEM disciplines and communication.

Another lesson plan with an 18-point difference between the structured and unstructured prompt evaluation results is Lesson Plan 13. The structured prompt AI evaluation describes the ‘Problem Identification’ phase as ‘clearly defining the engineering design process by focusing on real-world problems, addressing significant issues such as climate change and seed scarcity,’ and has awarded 3 points for this criterion. This scoring is due to the clear definition of the problem and providing a meaningful context to the students. In contrast, the unstructured prompt evaluation states, ‘The lesson plan effectively introduces a problem related to seed scarcity in a meaningful context that engages students, addressing real-world problems. This aligns well with the engineering design process by enabling students to understand local and global agricultural challenges,’ and has given full points. Regarding constraints and criteria, the structured prompt evaluation notes, ‘Constraints and criteria have been specified, but the processes for applying these constraints could have been more detailed,’ awarding 2 points to this area. No specific justification for this criterion has been observed in the other evaluation. A similar situation exists for science and mathematics outcomes. The evaluation using the structured prompt comments, ‘Outcomes are specified; however, more information is needed on whether the materials for measuring and implementing these outcomes have been fully developed,’ awarding 2 points to this category, indicating that the materials used may not have been fully developed. On the other hand, no evaluation for this category has been found using the unstructured prompt. In the multiple solutions category, the structured prompt describes, ‘The lesson plan allows students to develop multiple solutions.’ Conversely, the unstructured prompt negatively assesses this aspect, stating, ‘Students should be more encouraged to develop multiple solutions to problems, a fundamental element of the engineering design process.’

Another lesson plan showing a difference of 17 points between structured and unstructured prompt evaluation results is Lesson Plan 29. According to the structured prompt, this plan has been thoroughly evaluated for its alignment with the engineering design process and scored on each criterion. During the problem identification stage, fundamental information about the respiratory system was clearly articulated and the design problem was formulated, thus earning 3 points with the statement ‘The problem definition and issues to be resolved were clearly stated.’ Within the scope of constraints and criteria, safety measures and requirements of the design process were ‘clearly expressed,’ garnering another 3 points in this category. In terms of science and mathematics outcomes, the integration of respiratory system information with mathematical calculations ‘has helped reinforce both science and mathematics outcomes,’ earning full points for this evaluation. For clarity, the articulation of the design problem and outcomes ‘in a clear and understandable manner’ also secured full points in this category. Regarding student context, the relation of the lesson content to students’ lives and the support with real-life examples ‘have demonstrated how the learned information can be applied practically,’ resulting in 3 points. The categories of offering multiple solutions and research-inquiry were also scored 3 points each, as they provided opportunities for students to develop various solutions and to research topics in-depth. Criteria such as testability, integration with other STEM disciplines, and selection of the most suitable solution have successfully facilitated the lesson plan’s execution, with each receiving 3 points. The processes of prototype creation and testing, as well as communication, have imparted practical learning and effective presentation skills to students, hence both categories achieved full points. When the unstructured prompt AI evaluation is examined, the plan is specifically noted for ‘explaining the functions of structures and organs within the respiratory system through models’ and ‘discussing health precautions related to the respiratory system based on research.’ It is also remarked that the integration with mathematical calculations (calculations of circle and area of a circle) provides an interdisciplinary learning experience, thus encompassing Science and Mathematics outcomes. According to the unstructured prompt evaluation, the plan’s integration with the engineering and design process is accentuated by ‘expressing daily life problems or needs as design problems and including processes for developing solution proposals.’ Additionally, students were given ‘the opportunity to explain the basic stages of the design process and to discuss the production processes of design products.’ From an evaluation perspective, compared to the unstructured prompt, this lesson plan ‘comprehensively addresses the fundamental components of the engineering design process and includes activities that enable students to understand this process.’ However, ‘some aspects of the plan need to be developed further.’ Specifically, ‘the processes for applying constraints and criteria need to be more detailed, and the materials required for measuring and implementing outcomes need to be better developed.’ Nevertheless, as the unstructured prompt evaluation does not provide as detailed justifications as the structured prompt evaluation, it is unclear exactly where the evaluation scores are derived from. Therefore, a clear comparison in each category has not been possible.

The results of the study indicate that evaluations obtained using structured prompts offer higher consistency and specificity compared to those obtained using unstructured prompts. Specifically, it has been determined that when structured prompts are used, lesson plans more clearly and comprehensivelydefine the components of the engineering design process and this process is more effectively comprehended by students. Conversely, when unstructured prompts are used, lesson plans generally receive higher scores and there is a significantly lower agreement among evaluators. This situation suggests that unstructured prompts may be more susceptible to subjective interpretation and evaluation, and therefore, could create issues regarding consistency and standardization in the teaching process.

## Conclusion and discussion

The aim of this study is to determine the consistency between artificial intelligence and expert assessments in order to guide teachers’ processes of integrating engineering design phases into lesson plans. In this context, artificial intelligence systems utilizing both structured and unstructured prompts were compared with expert assessments. Based on the findings, best practices and recommended prompts are presented to help teachers more effectively incorporate engineering design phases into their lesson plans. The acceptance and rejection status of the research hypotheses based on the study findings is presented below.

Initially, the study compared evaluation scores from expert and structured prompt artificial intelligence in the assessment of teachers’ lesson plans, including the engineering design process. The findings revealed a high degree of consistency between expert evaluation scores and structured AI evaluation scores, with an intraclass correlation coefficient (ICC) of 70.8%. This significant level of agreement aligns with other studies that highlight AI tools’ ability to provide accurate and comprehensible recommendations [110]. For instance, AI models have been developed to assess disease severity in medical imaging, leading to improved consistency in predictions [111]. Similarly, AI language models like GPT-4 have shown potential in educational assessment processes, answering exam questions with high accuracy [112]. Despite this high agreement within the scope of the research, some inconsistencies were observed, indicated by a residual variance of 11.8. Upon investigating the reasons for this difference, it was found that expert evaluations were more critical and detailed, particularly in areas such as science, mathematics, research and inquiry, and prototype construction. The detailed and critical nature of expert evaluations, when considered within the framework of Bandura’s Social Cognitive Theory [113], reflects the influence of cognitively structured knowledge shaped by environmental experiences on the evaluation process. According to this theory, individuals’ information processing and decision-making are shaped by experiences acquired within social contexts. In this regard, experts are able to provide more detailed evaluations based on the cognitive schemas formed through their educational and professional experiences. In contrast, AI evaluations were generally more positive and holistic, offering an overall assessment of the current situation. The divergent evaluation approaches contributed to the observed score variance, with experts providing more nuanced critiques and AI providing broader acceptability assessments. The benefits of using AI tools in educational assessments include enhanced accuracy and efficiency [114]. This underscores the importance of integrating AI tools complementarily with expert oversight. Although structured prompts have current limitations, they can enhance the overall quality and effectiveness of educational assessments when used alongside human expertise.

In this research, the structured prompt encompassed all steps to represent the engineering design process as performance criteria. Structured prompts that specify performance criteria can lead to more accurate and consistent text production compared to unstructured prompts [80]. Additionally, the sophistication of the AI language model used in this study, specifically GPT-4, was noted. GPT-4 has been trained using both supervised and unsupervised learning techniques on extensive text data and fine-tuned through reinforcement learning with human feedback, making it a highly effective tool in educational settings [115]. GPT-4’s capabilities include reviewing and summarizing research papers, providing educational introductions to concepts, assisting in data interpretation, and offering feedback on written work when guided by well-structured prompts [116]. Its advanced natural language processing features enable computers to understand and generate human language, including tasks like language translation, semantic understanding, and information extraction from text-based data [117]. The high consistency between expert evaluations and structured prompt AI evaluations may be attributed to GPT-4’s natural language processing capabilities [118]. While the study demonstrates promising consistency between AI and expert evaluations, it is important to acknowledge that the AI model used (ChatGPT 4.5) is a general-purpose language model and not specifically trained for educational assessment tasks. This may limit its ability to produce nuanced or critical evaluations akin to those made by human experts.

Studies emphasize the importance of prompt formats in influencing the consistency and reliability of AI-generated responses [118,119]. In light of this, another research question addressed in this study was to determine the consistency between unstructured prompt AI evaluation scores and expert evaluation scores. The results of the ICC analysis revealed only a 7.6% agreement between the unstructured prompt AI evaluation scores and expert evaluation scores, and this level of agreement was not statistically significant. The Bland-Altman analysis indicated that the wide limits of agreement and systemic deviations between the two evaluation methods suggest that AI systems may yield significantly different results from expert evaluations. Other studies similarly indicate that the effectiveness of AI tools in providing accurate and reliable assessments can vary based on their application [120]. The low and non-significant correlation between unstructured prompt AI assessments and expert assessments, despite the high consistency between structured prompt AI assessments and expert assessments, underscores the differences in AI output depending on the prompt structure. Unstructured prompts can lead to more varied and unpredictable responses, affecting alignment with expert judgments [119].

This inconsistency may be explained through the lens of Cognitive Load Theory [121], which suggests that the absence of structure in prompts imposes a higher cognitive burden on the system. Unstructured prompts provide vague and under-specified input, thereby increasing the processing demands on the AI model. As a result, the system may struggle to organize relevant criteria or apply consistent judgment, leading to decreased alignment with expert evaluations. In contrast, structured prompts reduce extraneous cognitive load by clearly defining evaluation parameters, enabling the AI system to generate more coherent and consistent outputs.

The effectiveness of prompts in text production is crucial, as it impacts the quality and relevance of the generated text [81]. Given the lack of significant correlation between expert evaluation scores and unstructured prompt AI evaluation results, differences between structured and unstructured prompts were examined to determine their influence on the relevance of the produced text. The study found a 6.9% agreement between AI assessment scores derived from structured prompts and those from unstructured prompts, which was not statistically significant. This analysis indicates significant scoring discrepancies between the evaluators, with a low level of agreement. The Bland-Altman analysis further supported these findings, showing that structured prompt evaluation scores were on average 2.058 units lower than unstructured prompt evaluation scores. The discrepancy arose because unstructured prompt assessments provided more general evaluations, while structured prompt assessments offered detailed analyses, particularly in problem identification and criteria application processes. The findings suggest that structured prompt assessments yield lower scores due to detailed analysis, whereas unstructured prompts result in higher scores through a broader, more general evaluation approach. According to recent developments in prompt engineering research [93], structured prompts function as precise task definitions that narrow the model’s output space, thereby improving consistency and evaluation reliability. In contrast, unstructured prompts provide insufficient guidance regarding task scope and evaluation standards, which may lead to greater variation in AI responses and lower alignment with expert judgments. This phenomenon can also be interpreted from the perspective of Information Processing Theory. Structured prompts reduce cognitive ambiguity and provide a scaffold for how input is parsed and integrated by the AI system. Unstructured prompts, by contrast, may overwhelm or misguide the system due to their lack of specificity, resulting in inconsistent or superficial outputs.

This divergence in scoring patterns may also be attributed to optimism bias observed in AI-based evaluation systems, especially when dealing with vague or under-specified inputs such as unstructured prompts. In such cases, AI models tend to produce overly positive assessments, potentially overestimating the quality of lesson plans. This issue becomes particularly critical in high-stakes educational settings, where accuracy and reliability of assessment are paramount. Optimism bias can stem from the training data itself, which may carry inherent positivity biases or reflect idealized instructional content [122]. Otherwise, these systems risk reinforcing educational inequalities by creating new digital barriers in instructional evaluation.

The use of AI tools to design textual and visual materials and tasks accelerates teachers’ efforts to create engaging and comprehensive lesson plans [123]. AI facilitates the diversification of representation modalities in assessment practices, allowing teachers to save time and provide valuable feedback [124]. However, the effectiveness and reliability of AI tools in integrating engineering design processes into lesson plans and providing consistent assessments warrant careful consideration. This study’s findings, indicating high agreement between expert and AI judgments, suggest that AI can deliver reliable assessments consistent with expert evaluations. Furthermore, structured prompts offer greater consistency and authenticity compared to unstructured prompts. Consistent with the literature, structured prompts enhance the perceived quality of AI-generated responses [118], whereas unstructured prompts pose challenges in maintaining consistency and accuracy in evaluations [119]. Structured prompts appear to facilitate clearer and more detailed assessments of engineering design process components in lesson plans, while unstructured prompts tend to yield higher scores generally. This variability in evaluation may lead to inconsistencies and standardization issues in the teaching process, potentially providing misleading information for teachers integrating engineering processes into lesson plans. Although teachers are adept at planning standards-based lessons and integrating engineering practices, aligning these plans with both content and engineering design process standards can be complex and time-consuming [39]. Therefore, assessments using structured prompts can offer high-standard, consistent feedback for the engineering design process by evaluating the alignment of teachers’ lesson plans with engineering design process standards in a detailed and consistent manner. Caution is advised when using unstructured prompts in preparing lesson plans for the engineering design process, as they may lead to broader and more variable evaluation ranges.

This study examined the consistency between expert evaluations and artificial intelligence (AI)-based assessments of lesson plans incorporating engineering design processes. The findings indicate that AI, particularly when guided by structured prompts, can provide reliable and consistent evaluations that closely align with expert judgments. Structured prompts enable AI systems to generate detailed, criterion-referenced feedback, whereas unstructured prompts resulted in less consistent and more optimistic evaluations. The study further emphasizes that AI systems should not replace expert judgment but rather complement it. Expert evaluations remain indispensable for identifying the strengths and weaknesses of lesson plans in detail. At the same time, AI offers advantages in terms of speed, scalability, and providing initial feedback to support instructional design. Overall, the findings suggest that structured prompts present a promising approach for enhancing assessment practices.

### Future studies

The findings of this study offer valuable insights into how AI technologies can effectively support teachers in integrating engineering design processes into lesson plans. AI tools can assist teachers particularly in evaluating and improving lesson plans. However, the reliability of AI-based assessments is heavily influenced by the quality and structure of the prompts used. This underscores the importance of teachers’ critical thinking skills and pedagogical judgment in interpreting AI-generated feedback.

Based on the results, we recommend that teachers prioritize the use of structured prompts when seeking consistency, transparency, and alignment with specific pedagogical criteria. Structured prompts guide the AI in a more controlled and criterion-referenced manner, reducing ambiguity and enhancing reliability in evaluation outcomes. Furthermore, the structured prompt developed in this study can serve as a guiding framework for researchers and practitioners who wish to evaluate the extent to which lesson plans incorporate the engineering design process. Its alignment with validated instructional indicators makes it a practical tool for supporting curriculum development and instructional analysis. However, unstructured prompts may also have pedagogical value in exploratory or creative settings. They can help uncover innovative elements in lesson plans that may not be captured by rigid criteria, offering a broader perspective on instructional design. Therefore, the choice between structured and unstructured prompts should be aligned with the teacher’s goal—whether it is formative refinement, summative evaluation, or fostering creative thinking.

In practical terms, a hybrid approach—beginning with structured evaluation and then inviting unstructured insight—may provide teachers with both reliability and creative inspiration. We encourage future professional development programs to include training on prompt engineering to maximize the educational value of AI-assisted evaluation. While this study focused on evaluating the consistency between human and AI assessments, it did not examine the sensitivity of AI models to variations in instructional quality. Future research could stratify lesson plans by quality levels (e.g., top and bottom quartiles) to investigate whether AI tools can reliably distinguish between high- and low-quality instructional content.

### Limitations of the study

The limitations of this study stem from several key factors related to the scope of the tools and methods employed. First, only a single AI platform was used. Since results might vary with different algorithms and learning models, future studies should consider using multiple AI platforms to eliminate uncertainties about the reproducibility of the findings. Secondly, the study’s evaluation included only two types of prompts, limiting its scope and depth. Employing a variety of structured and unstructured prompts could provide a more comprehensive understanding of AI’s performance in evaluation processes and yield more reliable results across a broader dataset.

### Contributions of the study

This study contributes to the growing field of integrating artificial intelligence (AI) into educational assessment by demonstrating that AI can reliably evaluate lesson plans when supported by structured prompts. The findings highlight several key contributions:

#### Reliability of AI-based assessments.

The high agreement observed between expert and AI evaluations indicates that AI can serve as a consistent assessment tool, particularly when structured prompts are employed. This provides empirical support for the application of AI in instructional evaluation processes.

#### Importance of structured prompts.

The study underscores the critical role of prompt design. Structured prompts yielded more consistent and detailed evaluations aligned with expert judgments, whereas unstructured prompts produced overly optimistic and variable results. This distinction offers practical insights for educators and researchers into designing effective AI-assisted assessments.

#### Implications for teacher support.

By providing criterion-based and systematic feedback, structured AI evaluations can assist teachers in more effectively aligning their lesson plans with engineering design standards.

#### Caution against unstructured prompts.

The results caution against the use of unstructured prompts in high-stakes or standards-based contexts, as they may lead to inconsistencies and misguide teachers in instructional design decisions.

## Supporting information

S1 File Annex 1. **Example of Expert and Unstructured AI Evaluation Results for Lesson Plan 5.** Contains expert evaluation scores and justifications, along with unstructured prompt AI evaluation results for Lesson Plan 5, including detailed component-level assessments of the engineering design process.
(DOCX)

S2 File Structured and Unstructured Prompt AI Evaluation Results for Lesson Plan 5. Presents unstructured prompt AI evaluation results and structured prompt evaluation outcomes for Lesson Plan 5. Includes detailed scoring, category-based evaluations, and comparative analysis of engineering design process components.
(DOCX)
