# Correction: Comparative analysis of AI and expert evaluations in engineering design pedagogy

**DOI:** 10.1371/journal.pone.0338925

**Published:** 2025-12-12

**Authors:** Tuğra Karademir Coşkun, Esra Bozkurt Altan

There are errors in the author affiliations. The correct affiliations are as follows:

Tuğra Karademir Coşkun^1^, Esra Bozkurt Altan^2^

1 Department of Computer Education,Sinop University, Sinop, Türkiye, 2 Department of Science Education, Sinop University, Sinop, Türkiye
