# Supplementary material for: Comparative analysis of AI and expert evaluations in engineering design pedagogy
Source: PLoS One. 2025 Sep 22;20(9):e0332715. doi: 10.1371/journal.pone.0332715 (PMC12453255; doi:10.1371/journal.pone.0332715)
Supplement: S1 File — Example of Expert and Unstructured AI Evaluation Results for Lesson Plan 5. Contains expert evaluation scores and justifications, along with unstructured prompt AI evaluation results for Lesson Plan 5, including detailed component-level assessments of the engineering design process. (DOCX) [file pone.0332715.s001.docx]

LESSON PLAN 5 FROM THE EXPERT EVALUATION

***Lesson Plan 5 Evaluation:***

***Constraint: 3***
***Criteria: 3***
***Science/Mathematics Achievements: 2*** *The content partially aligns with the science achievements written in the lesson plan. It is recommended to ensure the content covers all achievements comprehensively.*

***Clarity: 1*** *The criteria and constraints are presented under separate headings in the problem. However, it is not clear what is expected from the students. Clearly state what you want the student to do.*

***Student Context: 3***
***Multiple Solutions: 3***
***Research-Inquiry: 3***
***Testable: 1*** *The lack of clarity in what is expected in the problem reduces testability. Develop a way to test based on what you want the student to design (e.g., create a rubric).*

***Other STEM Disciplines: 2*** *You can integrate technology or mathematics into your activity.*

***Development of Possible Solutions: 3***
***Selection of the Most Appropriate Solution: 2*** *You should provide opportunities for students to choose the most appropriate solution and reflect this in the activity. Consider adding techniques such as decision matrices or decision trees.*

***Prototyping and Testing: 1*** *Again, the lack of clarity in what is expected results in uncertainty about what to do and how to test it.*

***Communication: 1*** *Provide opportunities for students to present their designs and explain why their design is the best.*
