# Supplementary material for: Comparative analysis of AI and expert evaluations in engineering design pedagogy
Source: PLoS One. 2025 Sep 22;20(9):e0332715. doi: 10.1371/journal.pone.0332715 (PMC12453255; doi:10.1371/journal.pone.0332715)
Supplement: S2 File — Presents unstructured prompt AI evaluation results and structured prompt evaluation outcomes for Lesson Plan 5. Includes detailed scoring, category-based evaluations, and comparative analysis of engineering design process components. (DOCX) [file pone.0332715.s002.docx]

***Unstructured Prompt AI Evaluation Results: Lesson Plan 5:***

***Engineering Design Process Components***

1. ***Problem Identification (6/6)***
   - ***Detailed Problem Definition:*** *Yes*
   - ***Real-World Problem Association:*** *Yes*
   - ***Criteria and Constraints:*** *Yes*
   - ***Student Involvement:*** *Yes*
   - ***Ecological Problems and Solutions:*** *Yes*
   - ***Explanation:*** *The problem identification stage is quite detailed and comprehensive. It is associated with real-world problems and involves student participation. Criteria and constraints are clearly stated. The problem context is extensively addressed.*
2. ***Development of Possible Solutions (5/6)***
   - ***Research and Literature Review:*** *Yes*
   - ***Prototype Development and Testing:*** *Yes*
   - ***Group Work:*** *Yes*
   - ***Explanation:*** *The development of possible solutions stage is well-organized. The processes of research, literature review, and prototype development are thoroughly explained. Group work is effectively utilized. However, more technical details and case examples could be provided.*
3. ***Selection of the Most Appropriate Solution (4/6)***
   - ***Use of Decision Trees and Matrices:*** *Partially*
   - ***Evaluation of Solution Proposals:*** *Yes*
   - ***Use of Concepts:*** *Yes*
   - ***Explanation:*** *In the solution selection stage, the use of decision trees and matrices is mentioned but the detailed application part is missing. Apart from this, solution proposals are evaluated, and attention is given to students using concepts.*
4. ***Prototyping and Testing (5/6)***
   - ***Prototype Development:*** *Yes*
   - ***Testing and Improvement:*** *Yes*
   - ***Student Involvement and Evaluation:*** *Yes*
   - ***Explanation:*** *The prototyping and testing stage is detailed. Student participation and evaluation processes are mentioned. The testing and improvement stage is clearly explained. However, more testing and improvement cycles could be added.*
5. ***Communication and Presentation (6/6)***
   - ***Design Presentations:*** *Yes*
   - ***Evaluation and Feedback:*** *Yes*
   - ***Summary and Conclusion:*** *Yes*
   - ***Explanation:*** *Student presentations and evaluation stages are mentioned. The process summary and conclusion are done by the teacher. The communication and presentation part is effectively implemented.*

***Overall Evaluation and Scoring***

- ***Total Score:*** *26/30*
- ***Justification:*** *The activity plan generally covers the components of the engineering design process. The stages of problem identification, solution development, and prototyping are detailed and encourage student participation. The use of decision trees and matrices is partially mentioned; this part could be more detailed. The testing and improvement stages are clear. The communication and presentation part is effectively implemented.*

**Structured Prompt Used for Evaluating Lesson Plans:** *Please evaluate the following lesson plan for its alignment with engineering design processes. The evaluation should be conducted within the three specified categories ("Expected Level," "Acceptable," "Needs Improvement"). Each question is worth 3 points, with a maximum score of 36 and a minimum of 12. For each category, review the lesson plan according to the following criteria and assign an appropriate score for each criterion:*

*Engineering Design Process:*

1. *Problem Identification: Are the obstacles and necessary qualities for successfully designing the specified product or system clearly defined?*
2. *Constraint/Criterion: Is the process for evaluating criteria and constraints sufficiently clear?*
3. *Science/Mathematics Achievements: Are the materials necessary for measuring achievements developed, and are the implementation processes clearly defined?*
4. *Clarity: Are the design problem and expected achievements clearly and understandably expressed in the lesson plan?*
5. *Student Context: Is the problem situation appropriate for the students' social and cultural environment and related to real life?*
6. *Multiple Solutions: Does the lesson plan allow for the development of different solutions?*
7. *Research-Inquiry: Is the lesson plan prepared to support research and inquiry processes?*
8. *Testable: Is the planning and implementation process for testing the success of the design sufficiently detailed?*
9. *Other STEM Disciplines: Does the design problem include knowledge or skills from at least one STEM discipline?*
10. *Development of Possible Solutions: Is the research process and mini design tasks for the solution sufficiently defined?*
11. *Selection of the Most Appropriate Solution: Is the process of students deciding on a common design from different designs clearly expressed?*
12. *Prototyping and Testing: Is the process of prototyping and testing appropriately planned?*
13. *Communication: Are the processes for students to present and evaluate their designs in a classroom environment sufficiently detailed?*

*Evaluate each criterion, calculate the overall score of the lesson plan, and provide suggestions for improvement. List the total score and the individual scores for each question. Please review carefully before evaluating*.

**Structured Prompt AI Evaluation Results:**

***Lesson Plan 5:***

1. ***Problem Identification***
   - ***Evaluation:*** *Expected Level*
   - ***Score:*** *3/3*
   - ***Comment:*** *The activity plan effectively guides students in defining the necessary criteria and constraints for solving real-world problems. Detailed information is provided for problem identification and generating appropriate solutions.*
2. ***Constraint/Criterion***
   - ***Evaluation:*** *Expected Level*
   - ***Score:*** *3/3*
   - ***Comment:*** *The criteria and constraints are clearly defined, covering important areas such as sustainable energy use, economic factors, and environmental sensitivity.*
3. ***Science/Mathematics Achievements***
   - ***Evaluation:*** *Expected Level*
   - ***Score:*** *3/3*
   - ***Comment:*** *Science achievements, particularly in recycling, waste management, and sustainable energy, are well addressed. Mathematical calculations, including cost and measurement requirements, are also included.*
4. ***Clarity***
   - ***Evaluation:*** *Expected Level*
   - ***Score:*** *3/3*
   - ***Comment:*** *The lesson plan clearly presents the design problem and expected achievements.*
5. ***Student Context***
   - ***Evaluation:*** *Expected Level*
   - ***Score:*** *3/3*
   - ***Comment:*** *The activity is based on real-world problems relevant to the students' social and cultural environment, allowing them to establish a personal connection with the subject.*
6. ***Multiple Solutions***
   - ***Evaluation:*** *Expected Level*
   - ***Score:*** *3/3*
   - ***Comment:*** *Students are provided with the opportunity to explore various solution paths, which effectively fosters their creative thinking skills.*
7. ***Research-Inquiry***
   - ***Evaluation:*** *Expected Level*
   - ***Score:*** *3/3*
   - ***Comment:*** *Research and inquiry processes are emphasized throughout the activity, and various techniques are used to encourage active student participation.*
8. ***Testable***
   - ***Evaluation:*** *Expected Level*
   - ***Score:*** *3/3*
   - ***Comment:*** *Detailed planning for testing prototypes and solution proposals is provided, giving students the chance to evaluate their designs practically.*
9. ***Other STEM Disciplines***
   - ***Evaluation:*** *Expected Level*
   - ***Score:*** *3/3*
   - ***Comment:*** *The activity promotes an interdisciplinary approach, especially in environmental science and sustainable engineering.*
10. ***Development of Possible Solutions***
    - ***Evaluation:*** *Expected Level*
    - ***Score:*** *3/3*
    - ***Comment:*** *The solution development process is well-defined, allowing students to conduct research and test their hypotheses effectively.*
11. ***Selection of the Most Appropriate Solution***
    - ***Evaluation:*** *Expected Level*
    - ***Score:*** *3/3*
    - ***Comment:*** *The solution selection process guides students in evaluating different alternatives and choosing the most appropriate solution.*
12. ***Prototyping and Testing***
    - ***Evaluation:*** *Expected Level*
    - ***Score:*** *3/3*
    - ***Comment:*** *Prototyping and testing provide a realistic experience through hands-on learning, allowing students to assess their designs under real conditions.*
13. ***Communication***
    - ***Evaluation:*** *Expected Level*
    - ***Score:*** *3/3*
    - ***Comment:*** *The processes for groups to present and evaluate their designs in a classroom environment are sufficiently detailed, enhancing effective communication and presentation skills.*

***Overall Evaluation***

- ***Total Score:*** *36/36*
- ***General Comment:*** *The lesson plan comprehensively and effectively covers all stages of the engineering design process, allowing students to develop both their theoretical knowledge and practical skills.*

***Improvement Suggestions***

- ***No Areas for Improvement:*** *In its current form, the plan provides a comprehensive guide for students to understand and apply the engineering design process. However, adding more interdisciplinary activities could further strengthen students' connections with other STEM fields.*
